# Efficacy of the eribulin, pertuzumab, and trastuzumab combination therapy for human epidermal growth factor receptor 2–positive advanced or metastatic breast cancer: a multicenter, single arm, phase II study (JBCRG-M03 study)

**DOI:** 10.1007/s10637-020-00991-6

**Published:** 2020-08-24

**Authors:** Toshinari Yamashita, Hidetoshi Kawaguchi, Norikazu Masuda, Masahiro Kitada, Kazutaka Narui, Masaya Hattori, Tetsuhiro Yoshinami, Nobuki Matsunami, Kazuhiro Yanagihara, Teru Kawasoe, Takeshi Nagashima, Hiroko Bando, Hiroshi Yano, Yoshie Hasegawa, Rikiya Nakamura, Masahiro Kashiwaba, Satoshi Morita, Shinji Ohno, Masakazu Toi

**Affiliations:** 1 Department of Breast and Endocrine Surgery, Kanagawa Cancer Center, 2-3-2 Nakao, Asahi-ku, Yokohama, 241-8515 Japan; 2 Department of Breast Surgery, Matsuyama Red Cross Hospital, Matsuyama, Japan; 3 Department of Surgery, Breast Oncology, NHO Osaka National Hospital, Osaka, Japan; 4 Breast Disease Center, Asahikawa Medical University Hospital, Asahikawa, Japan; 5 Breast and Thyroid Surgery, Yokohama City University Medical Center, Yokohama, Japan; 6 Department of Breast Oncology, Aichi Cancer Center, Nagoya, Japan; 7 Department of Medical Oncology, Osaka International Cancer Institute, Osaka, Japan; 8 Department of Breast Surgery, Osaka Rosai Hospital, Sakai, Japan; 9 Department of Medical Oncology, Kansai Electric Power Hospital, Osaka, Japan; 10 Department of Breast and Endocrine Surgery, Japanese Red Cross Kumamoto Hospital, Kumamoto, Japan; 11 Department of General Surgery, Chiba University Hospital, Chiba, Japan; 12 Breast and Endocrine Surgery, Faculty of Medicine, University of Tsukuba, Tsukuba, Japan; 13 Department of Surgery, Nagasaki University Graduate School of Biomedical Sciences, Nagasaki, Japan; 14 Department of Breast Surgery, Hirosaki Municipal Hospital, Hirosaki, Japan; 15 Department of Breast Surgery, Chiba Cancer Center, Chiba, Japan; 16 Breast Surgery, Sagara Hospital, Kagoshima, Japan; 17 Department of Biomedical Statistics and Bioinformatics, Graduate School of Medicine Kyoto University, Kyoto, Japan; 18 Center of Breast Oncology, The Cancer Institute Hospital of JFCR, Tokyo, Japan; 19 Department of Surgery (Breast Surgery), Graduate School of Medicine Kyoto University, Kyoto, Japan

**Keywords:** Metastatic breast cancer, Chemotherapy, Anti-HER2 drug, Eribulin, Pertuzumab

## Abstract

*Purpose* To date, it is not clear which anticancer agent is useful in combination with trastuzumab and pertuzumab As the first and second selective regimens for advanced or metastatic breast cancer (AMBC), this multicenter, open-label, phase II trial (JBCRG-M03: UMIN000012232) presents a prespecified analysis of eribulin in combination with pertuzumab and trastuzumab. *Methods* We enrolled 50 patients with no or single prior chemotherapy for HER2-positive AMBC during November 2013–April 2016. All patients received adjuvant or first-line chemotherapy with trastuzumab and a taxane. The treatment comprised eribulin on days 1 and 8 of a 21-day cycle and trastuzumabplus pertuzumab once every 3 weeks, all administered intravenously. While the primary endpoint was the progression-free survival (PFS), secondary endpoints were the response rate and safety. *Results* Of 50 patients, 49 were eligible for safety analysis, and the full analysis set (FAS) included 46 patients. We treated 8 (16%) and 41 (84%) patients in first- and second-line settings, respectively. While 11 patients (23.9%) had advanced disease, 35 (76.1%) had metastatic disease. The median PFS was 9.2 months for all patients [95% confidence interval (CI): 7.0–11.4]. In the FAS, 44 patients had the measurable lesions and the complete response rate (CR) was 17.4%, and partial response rate (PR) was 43.5%. The grade 3/4 adverse events were neutropenia (5 patients, 10.2%), including febrile neutropenia (2 patients, 4.1%), hypertension (3 patients, 6.1%), and other (1 patient). The average of the left ventricular ejection fraction did not decline markedly. No symptomatic left ventricular systolic dysfunction was observed. *Conclusions* In patients with HER2-positive AMBC, eribulin, pertuzumab, and trastuzumab combination therapy exhibited substantial antitumor activity with an acceptable safety profile. Hence, we have started a randomized phase III study comparing eribulin and a taxane in combination with pertuzumab and trastuzumab for the treatment of HER2-positive AMBC. **Trial registration ID:** UMIN-CTR: UMIN000012232.

## Introduction

Gene amplification or the protein overexpression of human epidermal growth factor receptor 2 (HER2) is present in 15%–20% of breast cancer tumors [1]. Reportedly, HER2 is overexpressed in several cancer types and contributes to tumor cell proliferation, survival, differentiation, and migration [2–5]. Trastuzumab, a humanized monoclonal antibody, potently hinders the HER2-mediated signaling pathway and binds to domain IV of HER2. Pertuzumab is also a humanized monoclonal antibody that targets HER2 [6, 7]; however, unlike trastuzumab, it binds to domain II of the receptor and, thus, can disrupt HER2 dimerization and ligand-activated signaling with other growth factor receptors, including other HER family members. HER signaling warrants homo- or heterodimerization. Reportedly, the HER2-HER3 dimer is the most potent to induce cell proliferation [8–10].

In the clinical assessment of CLEOPATRA (clinical evaluation of docetaxel, pertuzumab, and trastuzumab), a double-blind randomized phase III trial comparing pertuzumab and trastuzumab+docetaxel with placebo+trastuzumab+docetaxel as the primary treatment for HER2-positive progressive and recurrent breast cancer, the response rates (RR) were 80.2% and 69.3% in the pertuzumab and control groups, respectively. In addition, the progression-free survival (PFS) was significant in the pertuzumab and control groups at 18.7 and 12.4 months, respectively; overall survival [OS; central mean observation period, 50 months; pertuzumab group, 56.5 months; control group, 40.8 months; hazard ratio (HR), 0.68; 95% confidence interval (CI): 0.56–0.84] was markedly prolonged in the pertuzumab group [11, 12]. The addition of pertuzumab did not increase cardiotoxicity or frequent adverse events (AE), except neutropenia (49% vs. 46%), febrile neutropenia (13% vs. 7%), and diarrhea (9% vs. 5%). We have concerns about the safety of the practical implementation of docetaxel for edema, peripheral neuropathy, and other AEs accompanied by a decline in the quality of life. Furthermore, the development of therapeutic methods that could exhibit equivalent effects and decrease side effects is a critical clinical problem regarding patients’ needs.

Reportedly, eribulin, a nontaxane-type microtubule dynamics inhibitor, inhibits tubulin polymerization, stops microtubule function, arrests G_2_–M phase cell cycle, and induces apoptosis [13–16]. The EMBRACE study markedly increased the primary endpoint of the OS with eribulin compared with the treatment of physician’s choice (TPC) in MBC patients. In the study, 762 patients with locally recurrent or metastatic breast cancer previously treated with 2–5 chemotherapeutic drugs, including anthracyclines and taxane anticancer drugs, were randomly allocated to the eribulin and TPC treatment groups, and the OS time was extended by 2.7 months [17]. The median PFS in the eribulin and TPC groups was 3.7 and 2.2 months, respectively. The EMBRACE trial included 16% of HER2-positive patients; even within that subset, eribulin exhibited good results over the entire survival period [17].

A phase II trial investigated a combination therapy of eribulin and trastuzumab as a major treatment for HER2-positive, locally advanced or metastatic breast cancer (AMBC) [18]. In the study, 22 (42.3%) of 52 patients had a history of anti-HER2 therapy; median treatment cycles were 10 cycles of eribulin and 11 cycles of trastuzumab, and the RR was 71.2% (*n* = 37), the median PFS was 11.6 months [18]. In this phase II study, the median PFS was approximately the same as 12 months of the trastuzumab and docetaxel groups in the CLEOPATRA trial. Grade 3/4 AEs were neutropenia (38.5%), peripheral neuropathy (26.9%), and fatigue (7.7%). These findings suggested that the combination of eribulin and trastuzumab is effective, well-tolerated, and could be one treatment option for HER2-positive locally AMBC. Hence, this trial aims to actively investigate the efficacy and safety as a phase II trial of the trastuzumab, pertuzumab, and eribulin combination therapy in HER2-positive breast cancer.

## Patients and methods

### Study design

We conducted this multicenter, single-arm, phase II study on patients with HER2-positive AMBC and assessed the efficacy and safety of eribulin in combination with trastuzumab and pertuzumab. This study was conducted per the tenets of the Declaration of Helsinki (2008), and the study protocol and informed consent were submitted for approval to the Institutional Review Committee of the participating institution. We obtained written informed consent from all patients before protocol treatment.

### Patients

In this study, patients who received first- or second-line therapy for HER2-positive AMBC were eligible. Patients within 12 months of completing perioperative chemotherapy and anti-HER2 therapy were treated as second-line treatment. The HER2-positive tumor was determined by score 3 on gene amplification by immunohistochemical staining or fluorescence in-situ hybridization based on the criteria of the 2013 American Clinical Oncology Association (ASCO/Recommendation of US pathologist (CAP) guideline [19]. In addition, all patients must have received taxanes and trastuzumab as adjuvant or recurrent therapy; previous hormonal therapy was accepted as well. All patients were eligible to assess tumor progression. Other eligibility criteria were as follows: aged 18–70 years with the expected survival time of >6 months, echocardiogram left ventricular ejection fraction (LVEF) at baseline ≥55%, Eastern Cooperative Oncology Group (ECOG) Performance status (PS) 0 or 1 (2 is allowed in case that the cause of decreased PS is bone metastasis), appropriate kidney, and bone marrow function.

Conversely, the exclusion criteria were as follows: symptomatic central nervous system metastasis, active systemic infection, prior use of eribulin, or recurrence in conserved breast or local recurrence, which is an appropriate treatment for reoperation.

### Treatment

All patients received 1.4-mg/m^2^ eribulin mesylate intravenously infused over 2–5 min on days 1 and 8 of each 21-day cycle. Pertuzumab and trastuzumab were administered to patients as follows: 840-mg pertuzumab (420 mg every 3 weeks), 8-mg/kg trastuzumab, and a maintenance dose of 6 mg/kg administered every 21 days. Of note, both pertuzumab and trastuzumab were administered intravenously over 90 min on day 1 of cycle 1; after that, it was injected over the course of 30 min on the first day of each cycle. This regimen was continued until the onset of progressive disease (PD) assessed by researchers based on the radiological evidence or until the onset of toxic effects that could not be managed effectively.

Notably, there was a decrease in the dose of eribulin, not pertuzumab and trastuzumab; two reductions (1.1 and 0.7 mg/m^2^) were allowed before stopping the study treatment or considering postponing the treatment cycle. If eribulin was discontinued owing to toxic effects or patients’ requirement after 6 cycles, pertuzumab and trastuzumab could continue. The use of prophylactic granulocyte colony-stimulating factor (G-CSF) was not allowed. If it is deemed necessary for patients’ welfare and is not expected to interfere with the evaluation of the clinical trial treatment, concomitant medication can be administered at the discretion of the investigator. No other antitumor therapy was permitted during the study treatment was ongoing.

### Endpoints

We performed the baseline tumor assessment (computed tomography or magnetic resonance imaging scan) of the chest, abdomen, pelvis, and other areas of known diseases 30 days before the first injection and every 9 weeks during treatment. In this study, the primary endpoint was the PFS, assessed by the investigators using RECIST ver.1.1 [20]. The secondary endpoints were the response rate (RR), safety, OS, efficacy among the patients after pre-use of pertuzumab, eribulin compliance, and efficacy of subsequent treatments. We defined RR as the percentage of patients who attained complete response (CR) and partial response (PR), and PFS as the duration from the date of study registry to the date of first confirming disease progression or death.

In addition, we performed the evaluation of electrocardiogram and echocardiogram at the baseline and every 4 cycles. Besides, laboratory examination of hematology and clinical chemistry was performed for each day of visit in the first cycle; after the second cycle, we managed to omit the laboratory test on the eighth day at the researcher’s discretion. We evaluated the ECOG PS in every visit. Moreover, AEs were rated on a 5-point scale based on the National Cancer Institute’s Common Terminology Criteria for Adverse Events version 4.0. All AEs were followed until resolution or 30 days after the patients’ last research visit. Notably, patients with SAE were followed until resolution of the event or stabilization of their condition. Nevertheless, AEs for onset peripheral neuropathy and any grade alopecia were followed until resolution or until initiation of another anticancer treatment. Since the Japanese version of PRO-CTCAE was not yet available during this study, side effects were assessed by the CTCAE evaluated by the physician.

### Statistical analysis

Based on the median of PFS of 9.2 months with trastuzumab+eribulin, 40% of patients received trastuzumab as preoperative and postoperative treatment, while 50% received a taxane or anthracycline [18]. In this study, we assumed the PFS for first and second line treatment after taxane and trastuzumab treatment as 6.0 months. Considering that the addition of pertuzumab results in 50% prolongation of the PFS based on the CLEOPATRA trial, we assumed the median expected PFS for the trastuzumab, pertuzumab, and eribulin combination therapy to be 9.0 months.

For a one-sided *α* error of 5%, detection power 80%, and accumulation of 2 and 3 years as a follow-up, 43 cases were necessary. Considering improperness and deviation, we set the enrollment as 48 cases. As the PFS varies depending on the therapeutic line, we made our final decision clinically based on the ratio of primary treatment and secondary treatment.

In this study, all efficacy analyses were primarily based on the full analysis sets (FAS), including all patients who received, at least, one study treatment. We analyzed the PFS using the Kaplan–Meier approach. As subgroup analysis, we evaluated a 95% CI and the median in the PFS for each group. A log-rank test with stratification by the pretreatment status was used to compare the PFS between each subgroup. Moreover, the Cox proportional hazards model was used to estimate HR with 95% CI according to stratification about previous treatment situations. We performed a prespecified subgroup analysis of independently assessed PFS to ascertain the consistency of the treatment effect based on the key baseline characteristics. Furthermore, AEs were assessed descriptively in a safety population (all patients who received, at least, one study drug administration). We used SPSS version 22.0 for windows (IBM Japan, Tokyo, Japan) for statistical analysis.

## Results

### Patients’ characteristics

Table 1 summarizes demographic and baseline clinical characteristics of the study cohort. We enrolled 50 patients in this trial from November 2013 to February 2016. One patient was dropped before the protocol treatment administration due to ineligibility, and 49 patients (median age: 56 years) were evaluated for safety. The FAS contained 46 patients. Of note, we excluded 3 patients from the FAS for the following reasons: (i) trastuzumab was not preceded; (ii) taxane was not preceded; and (iii) the interval from adjuvant therapy was too short for eligibility. The data blocking date for the effectiveness analysis was October 31, 2016, and the end date of safety analysis was at the completion of eight cycles for each patient.

**Table 1:** Patients’ characteristics

		Safety analysis group	First line	Second line
Number of patients		49 (100%)	8 (16%)	41 (84%)
Median age (range), years		56.0 (23–70)	56.0 (41–66)	56.0 (23–70)
ECOG performance status	0	36 (73%)	6 (75%)	30 (73%)
	1	13 (27%)	2 (25%)	11 (27%)
ER status	Positive	23 (47%)	5 (63%)	18 (44%)
	Negative	24 (51%)	3 (38%)	23 (56%)
PgR status	Positive	15 (31%)	3 (38%)	12 (29%)
	Negative	34 (69%)	5 (63%)	29 (71%)
HER2 status	IHC:3+	44 (90%)	8 (100%)	36 (88%)
	IHC:2+, ISH (positive)	5 (10%)	0	5 (12%)
Disease type at screening	Non-visceral	24 (49%)	3 (38%)	21 (51%)
	Visceral	25 (51%)	5 (63%)	20 (49%)
History of (neo)adjuvant trastuzumab		25 (51%)	8 (100%)	17 (41%)
History of (neo)adjuvant taxane		27 (55%)	8 (100%)	19 (46%)
Prior trastuzumab for metastasis		25 (51%)	0	25 (61%)
Prior pertuzumab for metastasis		12 (24%)	0	12 (29%)
Prior taxane for metastasis		23 (47%)	0	23 (56%)

ECOG, Eastern Cooperative Tumor Group; HER2, human epidermal growth factor receptor 2.ER, Estrogen receptor; PgR, Progesterone receptor; IHC, Immunohistochemical; ISH, In situ hybridazation

Of all 49 patients, 23 (47%) patients were estrogen receptor–positive, while 15 (31%) were progesterone receptor–positive. All patients with progesterone receptor–positive were estrogen receptor–positive. In addition, visceral metastasis was reported in 25 patients (51%). Previous treatment of trastuzumab was administered to all patients; 25 for (neo) adjuvant and 25 in the metastatic setting. Prior pertuzumab therapy was used in 12 patients (24%). Pretreatment T-DM1 was not an exclusion criterion. But there were no patients who used T-DM1 as perioperative or post-recurrence treatment. The median relative median dose of eribulin was 93.3% (range: 77%–100%).

### Efficacy

**Fig. 1:**
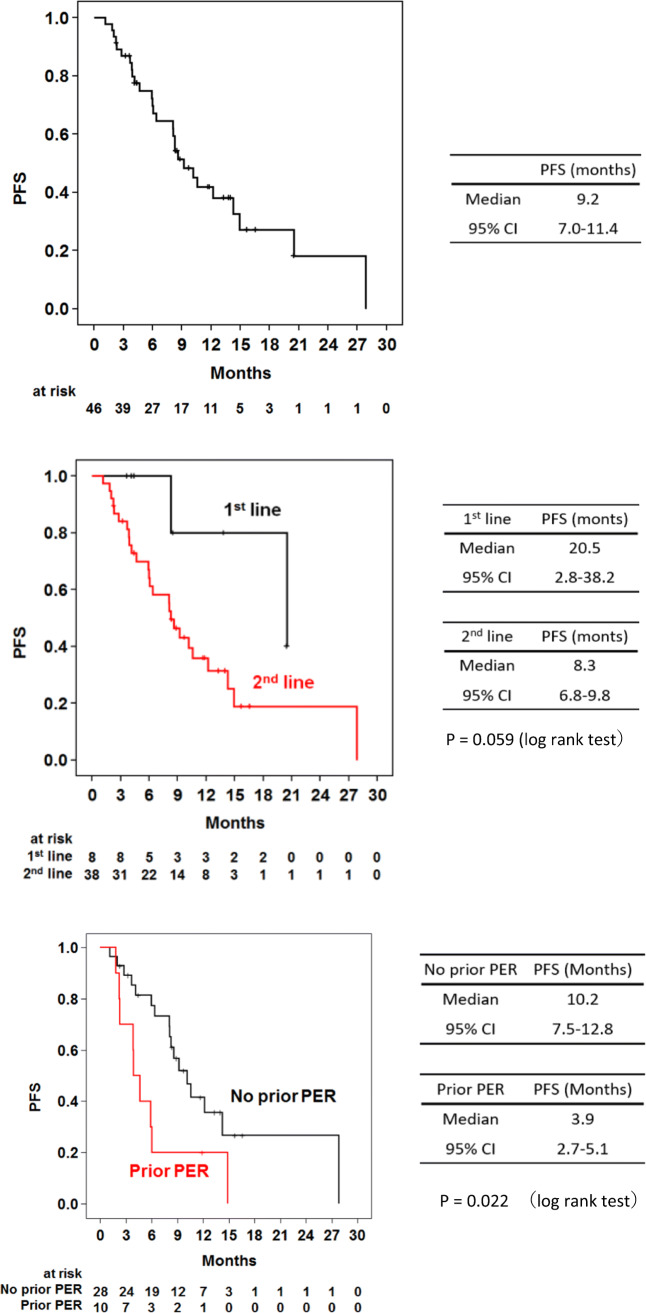
**a** Progression-free survival (PFS; full analysis set, FAS). **b** Progression-free survival (PFS; first line vs. second line). **c** Progression-free survival (PFS; no prior PER vs. prior PER in the second line)

Overall, the median PFS was 9.2 months for all patients (95% CI: 7.0–11.4; Fig. 1). Notably, the median PFS was 20.5 (95% CI: 2.8–38.2) months in the first-line treatment and 8.3 (95% CI: 6.8–9.8) months in the second-line treatment.

**Fig. 2:**
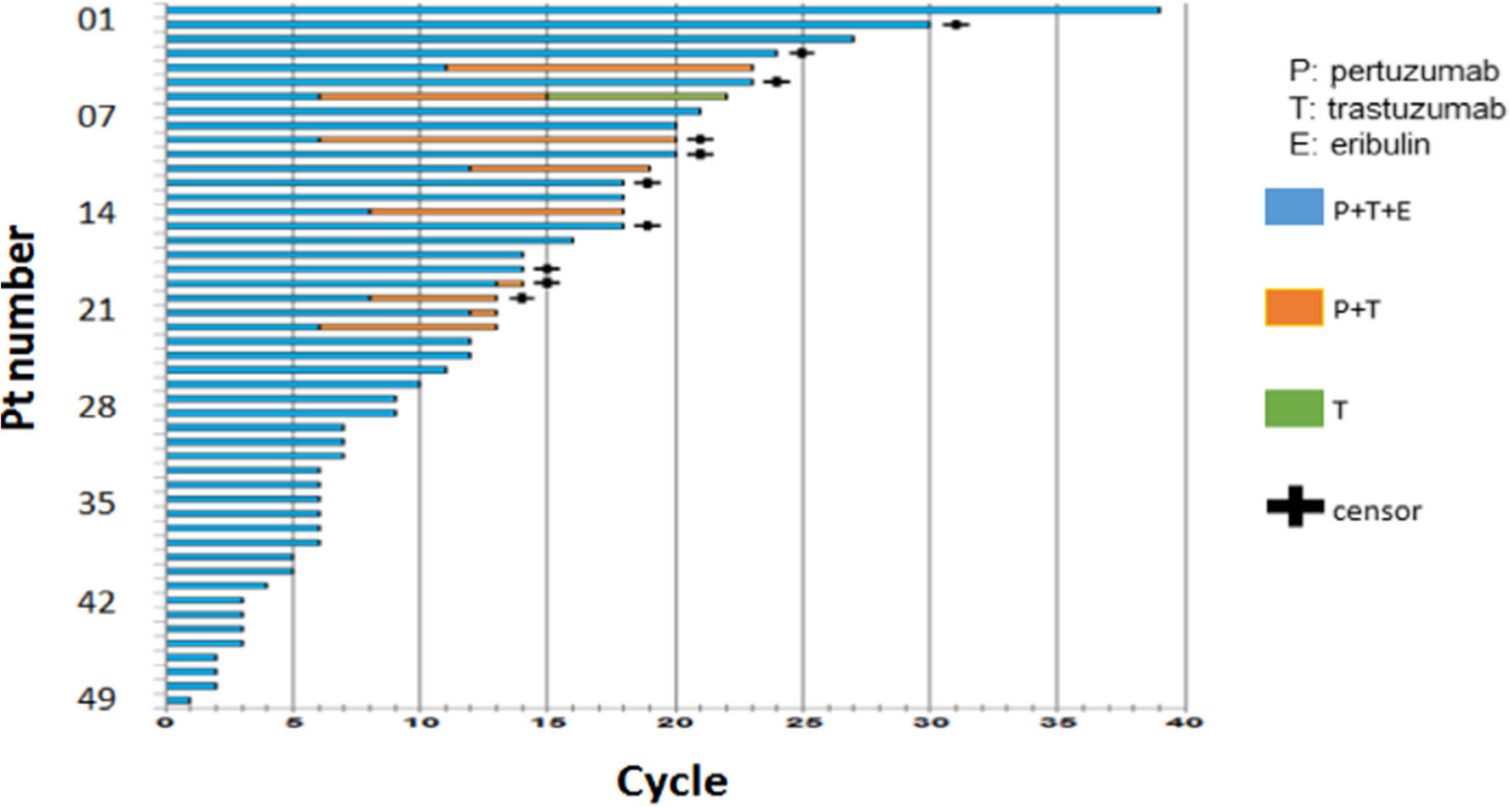
The Swimmer plot for tolerability

As the second-line treatment, the median PFS was 10.2 (95% CI: 7.5–12.8) months and 3.9 (95% CI: 2.7–5.1) months in patients treated without pertuzumab and with pertuzumab respectively. 46 patients in the full analysis set were evaluable for RR (60.9%; Table 2). Based on investigator assessment, the CR was 8 (17.4%), and the PR was 20 (43.5%). The RR of first-line treatment patients was 87.5% (Table 2), whereas it was 55.3% in second-line treatment patients. In particular, the RR of patients who received prior pertuzumab treatment was low; only 2 out of 10 achieved PR. The Swimmer plot revealed that 40 patients continued eribulin with trastuzumab and pertuzumab until disease progression or the blocking data point. By the time of drafting this manuscript, 10 patients were still ongoing treatment at this analysis (Fig. 2).

**Table 2:** Response rate

	FAS (*N* = 46)		First line (*N* = 8)		Second line (*N* = 38)	
	*N*	%	*N*	%	*N*	%
Response	28	60.9%	7	87.5%	21	55.3%
CR	8	17.4%	3	37.5%	5	13.2%
PR	20	43.5%	4	50.0%	16	42.1%
SD	11	23.9%	1	12.5%	10	26.3%
PD	5	10.9%	0	0%	5	13.2%
NE	2	4.3%	0	0%	2	5.3%

*FAS* full analysis set, *CR* complete response rate, *PR* partial response rate, *SD* stable disease, *PD* progressive disease, *NE* Not evaluable

### Safety

Table 3 lists the overall safety profile. All patients in this study reported treatment-related AEs. The hematological AEs with incidence >10% were leukopenia (14.3%), neutropenia (14.3%), and anemia (10.2%). The leading non-hematological AEs were peripheral neuropathy (34.7%), malaise (18.4%), alopecia (18.4%), nausea (12.2%), and appetite loss/diarrhea/mucositis/dysgeusia (10.2%). Furthermore, the leading grade 3/4 AEs were neutropenia/leukopenia (4.1%), febrile neutropenia (4.1%), peripheral neuropathy (2.0%), and appetite loss (2.0%,).

**Table 3:** Adverse events (AEs; incidence >10% or grade 3)

**Hematological**	**All (*N*** **= 49)**	**Grade ≤ 2**	**3 ≤ Grade**
Leucopenia	7 (14.3%)	5 (10.2%)	2 (4.1%)
Neutropenia	7 (14.3%)	2 (4.1%)	5 (10.2%)
Febrile neutropenia	2 (4.1%)	0	2 (4.1%)
Anemia	5 (10.2%)	5 (10.2%)	0
**Non-hematological**	**All (*N*** **= 49)**	**Grade ≤ 2**	**3 ≤ Grade**
Peripheral sensory neuropathy	17 (34.7%)	16 (32.7%)	1 (2.0%)
Malaise	9 (18.4%)	9 (18.4%)	0
Alopecia	9 (18.4%)	9 (18.4%)	0
ALT increased	6 (12.2%)	6 (12.2%)	0
Nausea	6 (12.2%)	6 (12.2%)	0
Appetite loss	5 (10.2%)	4 (8.2%)	1 (2.0%)
Diarrhea	5 (10.2%)	5 (10.2%)	0
Mucositis	5 (10.2%)	5 (10.2%)	0
Dysgeusia	5 (10.2%)	5 (10.2%)	0

**Fig. 3:**
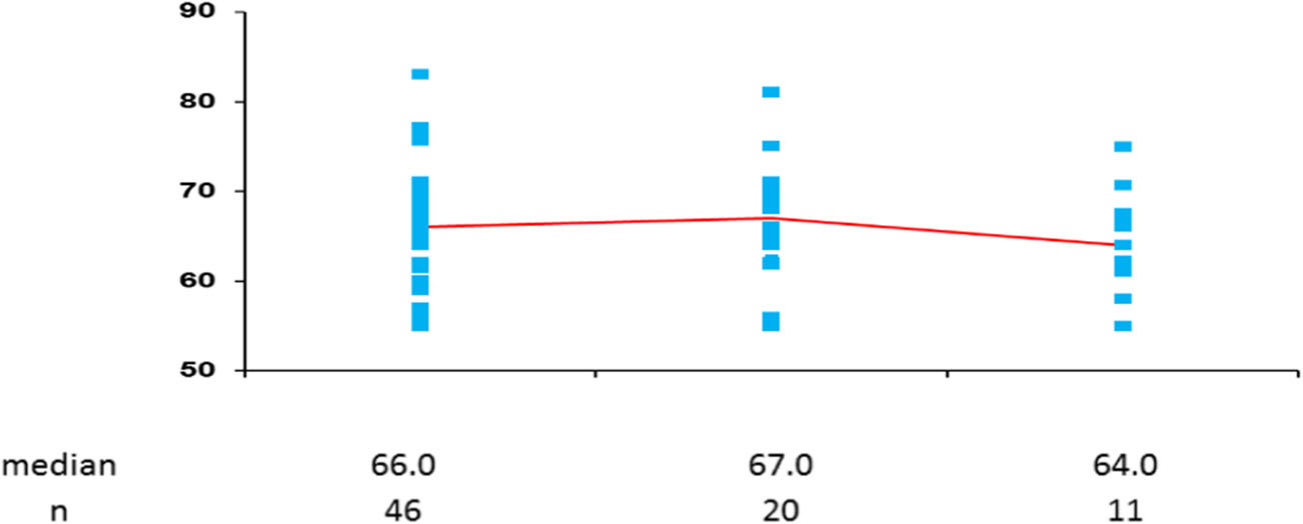
The ejection Fraction by UCG

In this study, we encountered 7 cases of serious AEs; 1 case was of interstitial pneumonia with recovery noted after 16 days. In addition, all cases of infusion-related reaction, febrile neutropenia, and vomiting recovered. Owing to the breast cancer progression, we observed one patient declined consciousness level, one patient bleeding from the liver, and one patient vomiting respectively. We did not observe asymptomatic left ventricular systolic dysfunction (includes asymptomatic LVEF drop of >10 percentage points below the baseline and value, 50%; symptomatic LVEF drop that required treatment or that led to treatment discontinuation) (Fig. 3).

## Discussion

From the CLEOPATRA study (*n* = 808), pertuzumab and trastuzumab+docetaxel is the standard therapy for patients with HER2-positive AMBC. In the phase II trial of pertuzumab and trastuzumab+paclitaxel as first- (*n* = 51) or second-line treatment (*n* = 69), the overall PFS was 19.5 (95% CI: 14–26) months, PFS was 24.2 (95% CI: 14 months–not reached) months and 16.4 months (95% CI, 8.5 months–not reached) for those without and with prior treatment, respectively [21]. In the study, grade ≥ 3 AEs were fatigue (6%), diarrhea, peripheral neuropathy, AST/ALT elevation, and limb syndrome (3%), skin dryness, and nausea (1.5%); no case of febrile neutropenia was noted. Overall, pertuzumab and trastuzumab+paclitaxel exhibited good efficacy and high tolerability. In preclinical data, vinca alkaloid vinorelbine demonstrated synergistic activity with trastuzumab against HER2-overexpressing breast cancer cells [22]. In HERNATA study, TTP (15.3 months vs. 12.4 months; HR: 094), OS (35.7 months vs. 38.8 months; HR: 1.01) was analogous to the study assessing trastuzumab+vinorelbine compared with trastuzumab+docetaxel as the primary treatment of HER2-positive advanced and recurrent breast cancer [23]. In this study, Median time to treatment failure for study chemotherapy was 5.6 months in the vinorelbine versus 7.7 months in the docetaxel, suggesting that vinorelbine may have achieved longer TTP. It is difficult to cure patients with recurrent breast cancer, and the purpose of treatment is to alleviate symptoms and prolong survival. Prolonged chemotherapy tends to have a longer prognosis [24], but docetaxel is difficult to continue after 8 cycles [11, 23]. Thus, vinorelbine was considered a candidate for combination with pertuzumab and trastuzumab. Reportedly, the median PFS was 14.3 months in the VELVET trial assessing vinorelbine+pertuzumab and trastuzumab treatment as the first-line treatment of HER2-positive AMBC [25].

In the EMBRACE trial, the OS time of eribulin-treated patients is longer compared with the TPC group [17]. Some studies have reported tumor blood vessel normalization and epithelial-to-mesenchymal transition (EMT) suppression as a new mechanism of action of eribulin [26, 27]. Reportedly, eribulin decreases TGF-β in tumors and plays a vital role in not only EMT but also the immune response in the tumor microenvironment [28–31]. In fact, in vivo experiments have demonstrated that the penetration of NK cells is induced by using eribulin [32]. Arguably, eribulin might prolong the OS by these mechanisms of action. Furthermore, a phase II trial investigated a combination therapy of eribulin and trastuzumab as a major treatment for HER2-positive, locally AMBC. All these findings indicate that the combination of eribulin and trastuzumab is effective, well-tolerated, and could be one treatment option for HER2-positive locally AMBC. Araki et al. [33] reported the safety and efficacy of eribulin, trastuzumab, and pertuzumab combination on 23 subjects as the third-line or later treatment; the combination therapy exhibited an acceptable safety profile, and the RR was 34.8%, which is favorable as a late-line therapy. To validate the efficacy of the eribulin, trastuzumab, and pertuzumab combination therapy on the front line treatment, our study was limited to primary and secondary treatments.

In our study, the eribulin, trastuzumab, and pertuzumab combination therapy exhibited many AEs, but most of them were not severe and their management was easy. The eribulin, trastuzumab, and pertuzumab combination therapy might be an altanative treatment of HER2-positive AMBC that has not previously used pertuzumab. In addition, this regimen could be an option if the avoidance of severe side effects is desirable in patients who have received taxanes pre- and postoperatively, Although the number of primary treatments was as small as 8 people, the median PFS exceeded 20 months in this study.

The limitation is that this trial is a single arm Phase 2 trial and we didn’t compare the eribulin, trastuzumab, and pertuzumab combination therapy with the standard therapy. Hence, we started a phase III trial (JBCRG M 06 study: NCT03264547) comparing eribulin and a taxane in combination with pertuzumab and trastuzumab for the treatment of HER2-positive AMBC [34].

## Conclusions

This study establishes that the first- and second-line treatment with the eribulin, pertuzumab, and trastuzumab combination therapy exhibits substantial antitumor activity with an acceptable safety profile in patients with HER2-positive AMBC. Hence, this study might provide evidence of the combined use of eribulin, trastuzumab, and pertuzumab as a new first- or second-line treatment for HER2-positive AMBC patients.
